# Dual biomarker role of PD-L1 and LC3B in glioblastoma: prognostic and therapeutic potential

**DOI:** 10.1007/s10143-025-04050-7

**Published:** 2026-01-24

**Authors:** Rana Fathy Torky, Rania Makboul, Dalia M. Badary, Wael M. A. El-Ghani, Ahmed El-Hakeem, Rabab M. H. El Ghorori

**Affiliations:** 1 Pathology Department, Faculty of Medicine, Assiut University, Al-Gamaa street, Assiut, Egypt; 2 Department of Neurosurgery, Faculty of Medicine, Assiut University, Assiut, Egypt; 3 Department of Pathology, Alazhar University, Cairo, Egypt

**Keywords:** GBM, PD-L1, LC3B

## Abstract

GBM, the most common primary malignant brain tumor in adults, has an overall dismal prognosis. Immunotherapy targeting the PD-1/PD-L1 axis has shown limited success in GBM. Resistance to therapies involves different pathways like autophagy. Detecting LC3B expression provides a simple technique for monitoring autophagy. Our goal was to understand the interplay between PD-L1 and LC3B in GBM prognosis and treatment strategies. The study analyzed 61 GBM specimens to assess the immunohistochemical expression of PD-L1 and LC3B with investigating their correlation with various clinicopathological parameters with assessing the impact of PD-L1 and LC3B expression on patients’ survival and the relation between both markers. Both PD-L1 and LC3B were significantly associated with clinicopathological parameters, including Karnofsky performance score (KPS)(*P* = 0.028 and 0.004 respectively), surgical resection extent (*P* = 0.023 and 0.002), treatment response(*P* = 0.015, *P* ≤ 0.001), patient outcome(*p* ≤ 0.001), and recurrence (*P* ≤ 0.001). There was a statistically significant inverse correlation between overall survival (OS) and both PD-L1 and LC3B expression. Additionally, there was a statistically significant inverse correlation between progression-free survival (PFS) and LC3B expression. PD-L1 expression, extent of resection and adjuvant chemotherapy were identified as independent prognostic factors for overall survival in GBM cases. A statistically significant positive relation existed between PD-L1 and LC3B (*P* ≤ 0.001). Results of this study suggest that the robust expression of PD-L1 in glioblastoma is associated with poor prognosis. Additionally, high expression of LC3B in GBM suggests increased autophagic activity which associated with unfavourable outcome. Combining immunotherapy with autophagy modulators could be a promising approach for improving GBM treatment.

## Introduction

 Glioblastoma, the most common primary malignant brain tumor, has a uniformly poor prognosis [1, 2]. Despite decades of research, GBM remains challenging to treat due to its rapid growth, diverse molecular characteristics, and ability to infiltrate vital brain structures [3]. Efforts are underway to extend GBM patients’ lifespan by understanding its molecular pathogenesis and exploring immunotherapy-based approaches [4]. Rational combinations of established treatments (such as programmed death-ligand 1(PD-L1)inhibitors) with other approaches may hold promise for better prognosis [5]. PD-L1(CD247), a crucial signaling pathway in tumor cells, influences various intrinsic functions and survival mechanisms, including autophagy [6]. Recent studies have highlighted the association between autophagy marked by high microtubule associated protein light chain 3Beta (LC3B) expression and unfavorable outcomes in different types of tumors [7]. To improve glioblastoma prognosis, researchers propose rational combinations of novel treatments, such as PD-L1 inhibitors, alongside approaches targeting autophagy inhibition [8]. Although PD-L1 and LC3B have each been investigated as individual biomarkers in glioblastoma, the interplay between immune evasion and autophagic pathways has not been systematically analyzed. To our knowledge, no previous study has evaluated their co-expression in glioblastoma or assessed their combined prognostic relevance. Our study therefore explores the dual biomarker potential of PD-L1 and LC3B, providing novel insights into the intersection between autophagy and immune modulation in GBM. To address this gap, our current work aims to evaluate PD-L1 and LC3B expression in GBM, explore their correlation with clinicopathological parameters, the relation between both markers and assess their impact on patients’ survival.

## Materials and methods

### Materials

Sixty one specimens of glioblastoma comprised the samples of this retrograde descriptive-analyticstudy. The samples were selected from surgical pathology laboratory at Assiut University Hospital, Assiut University, Egypt. The clinicopathological parameters and two years follow up data were obtained from Pathology and Oncology departments’ archive. Hematoxylin and eosin-stained slides for each case were reviewed by an expert pathologist to confirm the histopathological diagnosis. The clinicopathological characteristics of 61 GBM cases with details of the received treatment and midcyclic treatment response were summarized in Table 1; (Clinicopathologic parameters of the studied glioblastoma cases). This study is not a clinical trial. Clinical trial number: not applicable.

**Table 1:** Clinicopathologic parameters of the studied glioblastoma cases

Variables	No. (61)	%
Age: (years)		
< 50	26	42.6%
≥ 50	35	57.4%
Mean ± SD (Range)	48.92 ± 12.58 (19.0–70.0)	
Sex:		
Male	42	68.9%
Female	19	31.1%
Site of mass:		
Parietal	27	44.3%
Temporal	15	24.6%
Frontal	14	23.0%
Occipital	5	8.2%
Size of mass:		
Less than 5 cm	34	55.7%
More than 5 cm	27	44.3%
Lateralization:		
Right	36	59.0%
Left	25	41.0%
Karnofsky performance score		
≤ 60%	28	45.9%
> 60%	33	54.1%
Extent of surgical resection		
Complete resection	31	50.8%
Incomplete resection	19	31.2%
Biopsy	11	18.0%
Adjuvant chemotherapy:		
Yes	46	75.4%
No	15	24.6%
Number of cycles:		
< 6 cycles	27	58.7%
≥ 6 cycles	19	41.3%
Treatment response:		
Regression	15	24.6%
Stationary	34	55.7%
Progression	12	19.7%
2nd line chemotherapy:		
Yes	14	23.0%
No	47	77.0%
2nd line surgery:		
Yes	12	19.7%
No	49	80.3%
2nd line reirradiation:		
Yes	9	14.8%
No	52	85.2%

Data expressed as frequency (percentage), mean (SD), median

### Immunohistochemical methodology

A panel of programmed death-ligand 1 (PD-L1) and microtubule associated protein light chain 3 beta (LC3B) proteins was analyzed by immunohistochemical staining using the avidin-biotin immunoperoxidase complex technique following the manufacturer’s protocol. Tissue Sect. (4-µm thick) of formalin-fixed, paraffin-embedded specimens were cut. Sections were deparaffinized in xylene, rehydrated in graded alcohol, and transferred to PBS (phosphate-buffered saline, PH 6). The sections placed in an endogenous peroxide block for 15 min and For purpose of antigen retrieval for both PDL-1 & LC3B, sections were treated in microwave of (600 W) by immersion of the slides in citrate puffer solution (PH 7) for 20 min subsequently applied PD-L1 (mouse monoclonal antibodies, CUSABIO, Houston, USA, clone no.14D8C2, product code: CSB-MA878942A1m, 1: 200 [9]) and LC3B (mouse monoclonal antibody, CUSABIO, Houston, USA, Clone No. 18F2, Product Code CSB-MA171423, a dilution of 1: 100 for 60 min, and then immunocomplexes were visualized with diaminobenzidine for 10 min and covered by a coverslip. Finally, the slides were examined by Olympus light microscopy. Sections from tonsil were stained for PDL1 as positive control, with positive cytoplasmic staining in follicular macrophages (weak &moderate) and reticulated crypt epithelial cells (strong). Sections from placenta are stained as positive controls for LC3B with positivity was expressed as brownish cytoplasmic staining.

### Immunohistochemical evaluation

The slides were evaluated by 2 senior observers independently. PD-L1 expression was recorded according to the extent of diffuse/fibrillary and cytoplasmic staining throughout the tumor tissue, and was scored as a percentage of tumor cells expressing PD-L1into; high (≥ 50%), moderate: ((≥ 5% to < 50%), weak: (≥ 1% to < 5%), and none(0): (< 1)% of tumor cells are positive [10]. PD-L1 expression is categorized into: high (including high and moderate) and low including (weak) for statistical analysis. LC3B Immuonostaining was detected as cytoplasmic expression and, LC3B-stained tumor cells were scored in three 200× fields as: high LC3B expression: when above 50% of tumor cells are positive, low LC3B expression: when below 50% of tumor cells are positive [11].

### Statistical analysis

Results were statistically analyzed using SPSS software for Windows (version 20). All tests of significance were two sided and significance level in all used tests was (0.05). Quantitative characteristics of the group were presented as means and standard deviations (M ± SD) and compared with Student t test.

Categorical variables were implemented as frequency (percentage) and compared by using chi-square tests. Progression free survival and overall survival rates were performed using the Kaplan–Meyer method. Predictors of overall survival were determined by using logistic regression analysis. Level of confidence was kept at 95% hence, *P* value was significant if < 0.05.

## Results

The clinical characteristics and demographic data of 61 patients with glioblastoma were summarized in Table 1. The age of patients ranged from 19 to 70 years with mean: 48.92 years ± 12.58 Out of 61 cases, 42 were males and 19 were females. According to KPS: 28 patients had KPS of ≤ 60%, while 33 had KPS of > 60%. Regarding the tumor site; 27 were parietal, 15 temporal, 14 frontal and 5 occipital. Thirty six were right sided and 25were left sided. In 34 cases the tumor size was less than 5 cm and in 27 cases was more than 5 cm. As regards the surgical procedure, 31 achieved GTR whereas 19 achieved STR and 11 had only tumor biopsy.

Regarding the received treatment Most patients (*n* = 46) received adjuvant chemotherapy. Among them, 27 patients completed fewer than six cycles, while 19 patients received six or more cycles. During follow-up, 34 patients demonstrated a stationary disease course, 12 showed disease progression, and 15 experienced a regressive course. A total of 14 patients (23%) received second-line chemotherapy. In addition, 12 patients underwent second-line surgery, and 9 patients received second-line radiotherapy.

### Expression of PD-L1 and LC3B and their correlation with the clinicopathological parameters

PD-L1 immunoreactivity in GBM specimens demonstrated a diffuse/fibrillary cytoplasmic pattern. Cases were classified as having high (31/61; 50.8%), moderate (8/61; 13.1%), and weak (22/61; 36.1%) PD-L1 expression. For statistical purposes, PD-L1 expression was dichotomized into high (high + moderate; *n* = 39) and low (weak; *n* = 22).

**Fig. 1:**
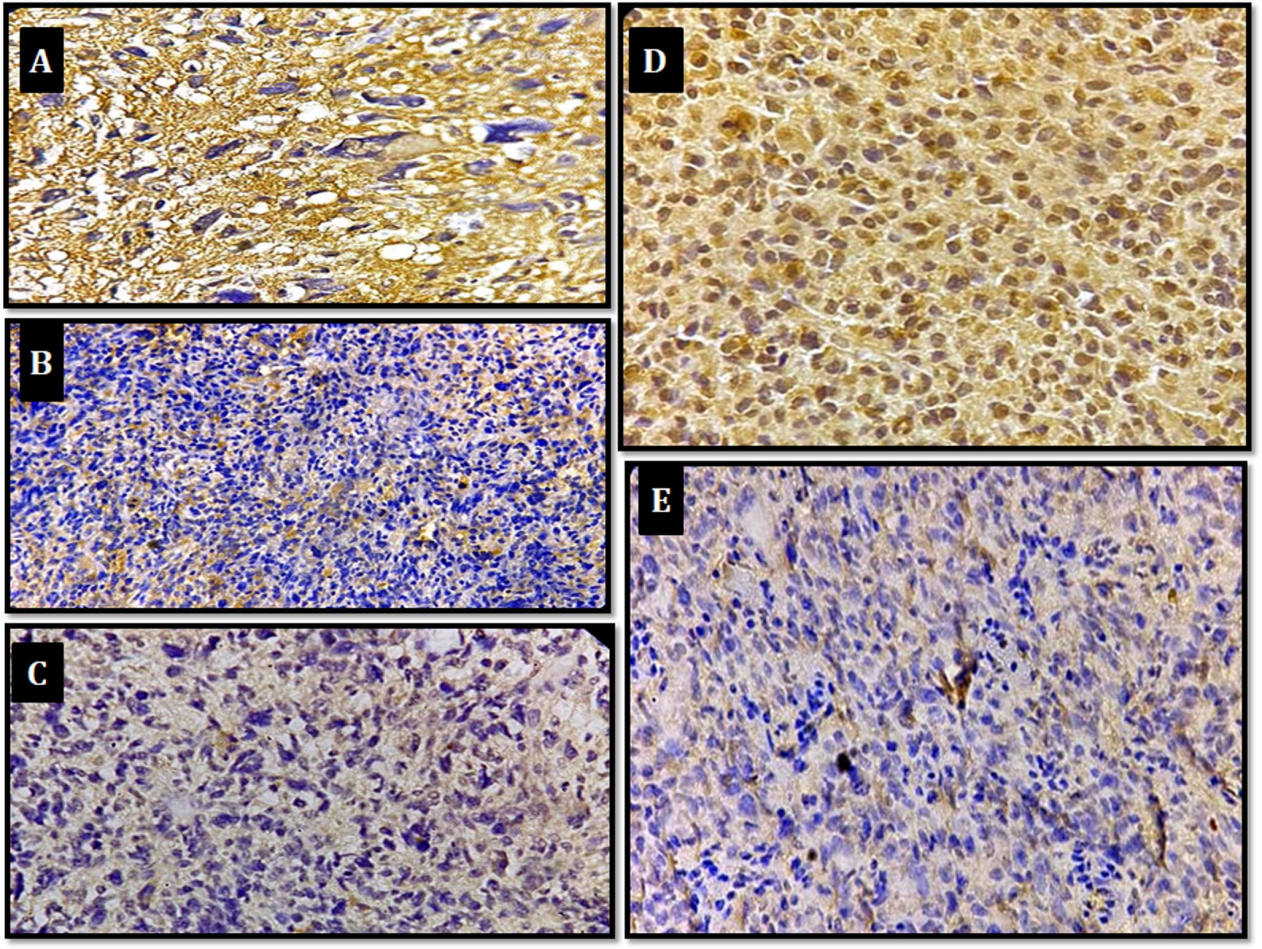
**(A)** Diffuse/fibrillary strong PD-L1 expression in glioblastoma (IHC, x400). **(B)** Diffuse/fibrillary PD-L1 moderate expression in glioblastoma (IHC, x200). **(C)** Diffuse/fibrillary and cytoplasmic weak PD-L1 expression in glioblastoma (IHC, x400). **(D)** High LC3B cytoplasmic expression in glioblastoma (IHC, x400). **(E)** Low LC3B expression in glioblastoma (IHC, x200)

LC3B showed cytoplasmic expression in all samples, with high expression observed in 27 patients (44.3%) and low expression in 34 patients (55.7%), as presented in Table 2; Fig. 1.

**Table 2:** Expression of PD-L1 and LC3B in GBM specimens and their association with different clinicopathological parameters

	PD-L1				P-value	LC3B				P-value
	High (Moderate & High)		Low (Weak)			High		Low		
	No.	%	No.	%		No.	%	No.	%	
Age (years)										
<50	13	50.0	13	50.0	0.051	10	28.5	16	61.5	0.432
≥ 50	26	74.3	9	25.7		17	48.6	18	51.4	
Sex:										
Male	27	64.3	15	35.7	0.932	21	50.0	21	50.0	0.180
Female	12	63.2	7	36.8		6	31.6	13	68.4	
Karnofsky performance score:										
≤ 60%	22	78.6	6	21.4	0.028*	18	64.3	10	35.7	0.004*
> 60%	17	51.5	16	48.5		9	27.3	24	72.7	
Tumor Site:										
Parietal	16	59.3	11	40.7	0.639	10	37.7	17	63.0	0.730
Frontal	11	78.6	3	21.4		7	50.0	7	50.0	
Temporal	9	60.0	6	40.0		8	53.3	7	46.7	
Occipital	3	60.0	2	40.0		2	40.0	3	60.0	
Size of mass:										
Less than 5 cm	21	61.8	13	38.2	0.692	17	50.0	17	50.0	0.311
More than 5 cm	18	66.7	9	33.3		10	36.0	17	63.0	
Lateralization										
Right	21	58.3	15	41.7	0.274	13	36.1	23	63.9	0.124
Left	18	72.0	7	28.0		14	56.0	11	44.0	
Extent of surgical resection										
Complete resection	15	48.4	16	51.6	0.023*	7	22.6	24	77.4	0.002*
Incomplete resection	14	73.7	5	26.3		13	68.4	6	31.6	
Biopsy	10	90.9	1	9.1		7	63.6	4	36.4	
Adjuvant chemo-therapy:										
Yes	28	60.9	18	39.1	0.383	19	41.3	27	58.7	0.415
No	11	73.3	4	26.7		8	53.3	7	46.7	
Number of cycles:										
< 6 cycles	19	70.4	8	29.6	0.116	10	37.0	17	63.0	0.483
≥6 cycles	9	47.4	10	52.6		9	47.7	10	52.6	
Treatment response:										
Regression	8	53.3	7	46.7	0.015*	4	26.7	11	73.3	≤0.001*
Stationary	19	55.9	15	44.1		11	32.4	23	67.6	
Progression	12	100.0	0	0.0		12	100.0	0	0.0	
2^nd^line chemo-therapy										
Yes	9	64.3	5	35.7	0.975	9	64.3	5	35.7	0.086
No	30	63.8	17	36.2		18	38.3	29	61.7	
2^nd^line surgery:										
Yes	6	50.0	6	50.0	0.322	6	50.0	6	50.0	0.655
No	33	67.3	16	32.7		21	42.9	28	57.1	
2^nd^line re-irradiation:										
Yes	6	66.7	3	33.3	1.000	4	44.4	5	55.6	1.000
No	33	36.5	19	36.5		23	44.2	29	55.8	
Outcome:										
Alive	15	42.9	20	57.1	≤0.001*	8	22.9	27	77.1	≤0.001*
Dead	24	92.3	2	7.7		19	73.1	7	26.9	
Recurrence:										
Yes	21	100.0	0	0.0	≤0.001*	19	90.5	2	9.5	≤0.001*
No	8	45.0	22	55.0		8	20.0	32	80.0	

*Significant, Using Percentage of Row

Chi-square test

Fisher Exact test

Significant associations were identified between PD-L1 and LC3B expression and Karnofsky Performance Status (*P* = 0.028 and *P* = 0.004, respectively). Both markers also correlated with the extent of surgical resection (PD-L1: *P* = 0.023; LC3B: *P* = 0.002). High PD-L1 expression was predominantly observed in biopsy-only procedures (90.09%). In contrast, low LC3B expression was more frequently detected among patients who underwent complete tumor resection (24/31; 77.4%), whereas high LC3B expression was more prevalent in patients with incomplete resection and biopsy (68.4% and 63.6%, respectively).

A statistically significant association was further noted between the expression of both markers and treatment response (PD-L1: *P* = 0.015; LC3B: *P* ≤ 0.001). All patients exhibiting disease progression (*n* = 12) demonstrated high expression of both PD-L1 and LC3B. Tumor recurrence also showed a strong association with biomarker expression (*P* < 0.001); 19 of 21 recurrent cases displayed concurrent high PD-L1 and LC3B expression, while two cases exhibited high PD-L1 with low LC3B expression. Moreover, PD-L1 and LC3B expression were significantly associated with overall patient outcome (*P* < 0.001).

No significant associations were observed between PD-L1 or LC3B expression and patient age, sex, tumour characteristics (location, lateralization, or size), presence or absence of adjuvant chemotherapy or number of adjuvant chemotherapy cycles, second-line chemotherapy, second-line surgery, or second-line radiotherapy (*P* > 0.05), as summarized in Table 2.

### Association between PD-L1 and LC3B expression

There was a statistically significant association between PD-L1 and LC3B expression (*P* < 0.001). Most of cases 25(92.6%) with high LC3B expression had high PD-L1expression as shown in Table 3.

**Table 3:** Association between LC3B and PD-L1 expression in the studied specimens:

PD-L1	LC3B				P-value
	High (*n* = 27)		Low (*n* = 34)		
	No.	%	No.	%	
High(high and moderate)	25	92.6%	14	41.2%	≤ 0.001*
Low(weak)	2	7.4%	20	58.8%	

Fisher Exact test Column percentage statistical significant difference if *P* < 0.05

### Survival analysis

#### PD-L1 and LC3B correlation with overall survival

**Fig. 2:**
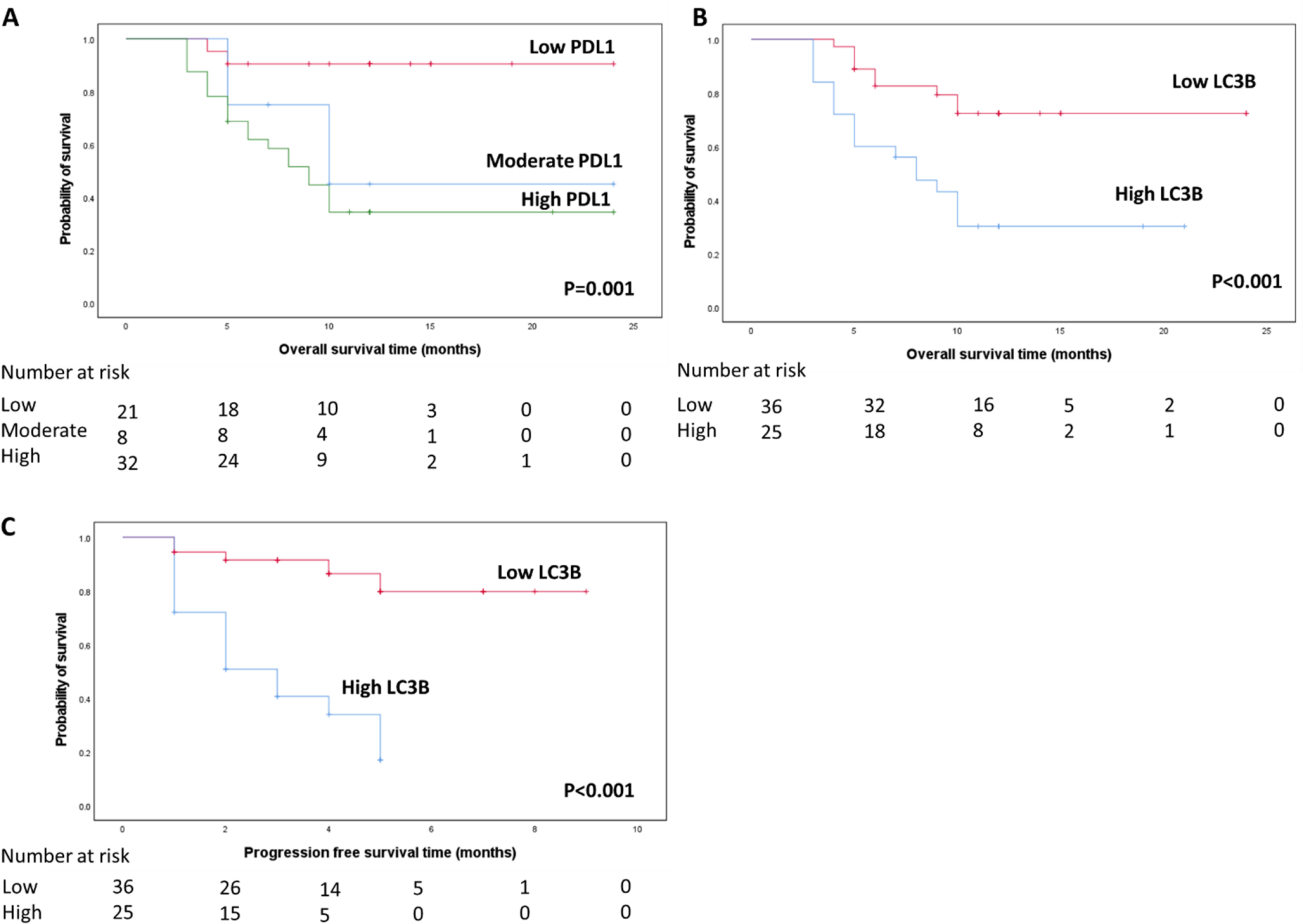
OS according to expression of each PD-L1& LC3B and PFS according to LC3B; (**A**) OS in patients with high PD-L1 expression= 8.07 ± 0.62(Mean±SE) with 95% C.I (6.85-9.281), moderate expression OS= 9.65 ± 1.00 with 95% C.I (7.68-11.62) and weak PD-L1 expression OS=23.14 ±1.26with 95%C.I (20.67-25.60). (**B**) OS in cases with high LC3B expression=9.88 ± 1.19 (Mean±SE) with 95%C.I (7.55-12.21), and in those with low expression =20.77 ± 1.42 with 95%C.I (17.99-23.56). (**C**) PFS in cases with High LC3B expression = 2.93 ± 0.33(Mean±S.E )with 95%C.I=2.27-3.58. PFS in cases with Low expression =8.47 ± 0.36 with 95%C.I =7.75-9.18

A statistically significant inverse correlation was observed between overall survival (OS) and PD-L1 and LC3B expression. Patients with high PD-L1 expression had significantly shorter OS compared with those with moderate and weak expression (8.07 vs. 9.65 vs. 23.14 months, respectively; *P* = 0.001). Similarly, high LC3B expression was associated with the lowest OS (9.88 ± 1.19 months), whereas patients with low LC3B expression had higher OS (20.77 ± 1.42 months), as shown in Fig. 2A–B.

Progression-free survival (PFS) also demonstrated a statistically significant inverse correlation with LC3B expression (*P* < 0.001). Patients with low LC3B expression exhibited a lower recurrence rate and significantly higher PFS (Fig. 2C).

#### Cox regression analysis of prognostic factors for overall survival

Multivariate Cox regression analysis was performed including several prognostic parameters. PD-L1 expression emerged as an independent prognostic factor for OS in GBM patients. High PD-L1 expression was associated with increased mortality (hazard ratio [HR] = 9.5; *P* = 0.02). Other independent prognostic factors included the extent of surgical resection (HR = 0.21; *P* = 0.008) and adjuvant chemotherapy (HR = 0.26; *P* = 0.02), as summarized in Table 4.

**Table 4:** Cox regression analysis of prognostic factors for overall survival:

	*P*-value	Hazard ratio	95.0% CI	
			Lower	Upper
PDL (high vs. low)	0.02*	**9.5**	1.3	67.8
LC3B (high vs. low)	0.15	2.2	0.76	6.7
KPS (> 60% vs. ≤ 60%)	0.11	2.5	0.80	8.13
Extent of resection (resection vs. biopsy)	**0.008**	**0.21**	0.07	0.67
Age (≥ 50 vs. < 50 years)	0.66	1.3	0.43	3.8
Size of mass (≥ 5 cm vs. < 5 cm)	0.70	0.80	0.27	2.4
Adjuvant chemotherapy	0.02*	**0.26**	0.08	0.80
2nd surgery	0.05	8.3	0.98	69.4
2nd reirradiation	0.47	0.48	0.07	3.4

Statistically significant difference if *P* < 0.05

## Discussion

Glioblastoma is the most common and aggressive primary malignant brain tumor in adults. It was considered a type of “immunologically cold tumor” due to the relative lack of tumor infiltrating T cells in the tumor micro-environment and high selectivity of blood brain barrier and activation of various immune escape mechanisms including programmed cell death ligand 1 [12]. Programmed death-ligand 1 contributed to immune evasion via binding to the programmed cell death protein-1 of immune cells, resulting in the inhibition of T cell functions and immune escape [13]. The “cold” phenotype of GBM may thus limit immunotherapy efficacy. Given the limited benefit to date with single agent PD-L1 blockade, combination therapies are now being pursued in GBM patients [14]. Combinational treatment approaches may help shift the “cold” microenvironment and enhance response to immune checkpoint blockade in GBM, including radiation therapy, chemotherapy, other immunotherapies, and autophagy modulators [15]. PD-L1 regulate many other intrinsic cell functions, including autophagy modulation [16]. LC3B is widely used as an autophagy marker and its role in tumors is an interesting area of research [17–19]. Several recent studies reported that autophagy and high LC3B expression are associated with poor prognosis and treatment outcome in various tumors [20–22].

There is paucity in literature about the dual role of PD-L1 an LC3B expression in GBM. The current study was conducted on 61 specimens of GBM patients aimed to evaluate PD-L1 &LC3B expression in glioblastoma besides their correlation with different clinicopathologic parameters, and the relationship between their expressions and to assess the effect of PD-L1 &LC3B expression on survival analysis of patients with GBM.

In this study, PD-L1 expression showed predominantly cytoplasmic, diffuse/fibrillary staining pattern and not membranous expression. These results were consistent with previous studies [23–25]. Berghoff et al. stated that a prominent diffuse/fibrillary and cytoplasmic PD-L1 immunostaining pattern of variable extent was seen throughout the tumor tissue. They believed it likely relates to membrane-bound PD-L1 on the delicate and intermingled tumor cell processes that form the pathognomic neurofibrillary matrix of diffuse astrocytic gliomas [4]. In addition, this diffuse cytoplasmic staining is related to internalized surface PD-L1 molecules, as PD-L1 storage and degradation in lysosomes have been described in lymphoma model [26, 27].

The main finding in this study is that all specimens had positive expression for PD-L1 similar to other studies [28, 29]. We found high PD-L1 expression in (50.8%), moderate expression in (13.1%) and weak expression in (36.1%) which was in agreement with the results of previous studies [25, 30]. In the current study there was negative significant statistical association (*P* = 0.028) between PD-L1 expression and KPS. To our knowledge no study investigated the association between PD-L1 expression and KPS but as other studies found significant correlation between PD1 expression and PD-L1 expression [31, 32], our results could be explained by the results of Romagnoli et al., who stated that; high PD1 expression was inversely associated with KPS [33].

In this study, all patients with recurrence exhibited high PD-L1 expression with statistically significant association (*P* < 0.001). However this observation is based on a limited sample size and should therefore be interpreted with caution. Among cases without recurrence (45%) had high PD-L1 expression and (55%) had low PD-L1 expression. In agreement with the study of Yu et al., who found more PD-L1 expression in recurrent than primary gliomas(74% vs. 33%respecively) [34].

Also, there was a highly positive statistically significant association between PD-L1 expression and patients’ outcome PD-L1 showed statistically significant inverse association with OS (*P* < 0.001); patients with high PD-L1 expression had shorter OS than those with low expression. This was in line with the other studies [4, 25, 35, 36]. However, this was contrasted with some studies [37, 38]. An increasing number of researchers have performed studies aimed to investigate the prognostic value of PD-L1 in GBM, but the results so far have been controversial with inconsistent results across various tumor types including GBM [39, 40]. Authors speculate that the number of CD8 + TILs is increased in PD-L1-positive cases, and that was the reason why patients with PD-L1-positive tumors had a good prognosis. On the contrary, other studies suggested that tumor cells might increase the expression of PD-L1, and upon interaction with PD-1 produced by activated T lymphocytes, tumor cells escape from immune destruction [41]. In our study, PD-L1 expression was significantly associated with adverse prognosis. The discrepancy in PD-L1 prognostic role can also be explained by several factors such as; the use of different types of antibodies, diverse conditions for immunohistochemistry including different diagnostic standards (e.g., expression patterns and positivity cutoffs), and technical issues. Additionally the conflict and controversial results could be attributed to the fact that the detection of PD-L1 expression in tumors is affected by spatial factors (heterogeneity of PD-L1 expression within the tumor and between different tumor sites) and temporal factors (hetrogenity of PD-L1 expression from before to after chemotherapy) leading to erroneous interpretation of the results [42, 43]. PD-L1 expression has also been linked to the mesenchymal GBM subtype and sarcomatous dedifferentiation, suggesting that it contributes to both immune evasion and tumor-intrinsic aggressiveness through EMT-related pathways [44].

A promising preclinical data in murine models of glioma have provided support for PD-L1 checkpoint inhibitors implementation in GBM patients [45, 46]. However using single immune checkpoint inhibitors have not demonstrated any significant efficacy in GBM treatment, so combination therapies are now being pursued [15, 47, 48]. Autophagy inhibitors have been proven efficacious in various cancers and their combinations with other treatment modalities including immune checkpoints inhibitors could provide better therapeutic response and increase therapeutic efficacy [47]. LC3B is widely used as an autophagy marker and its role in tumors is an interesting area of research [17–19].

In the current study there was a statistical significant association between LC3B and KPS (*P* = 0.04) which agrees with the study of Aoki et al. which found similar significant statistical interaction between KPS and LC3B expression (*P* = 0.041) [19].

There was significant inverse relation between LC3B expression and recurrence & outcome (*p* ≤ 0.001). Patients with low expression had better outcome with low recurrence and significantly higher OS and PFS (*P* < 0.001) which was in agreement with other studies [11, 17, 49]. Jiang and Wu stated that glioma cells that promote autophagy under adverse circumstances to sustain their survival, also evade the physiological response to cancer and therapy with the consequent tumor recurrence and progression [11]. Furthermore, autophagy allows the maintenance of glioma stem-like cells, which induces therapeutic resistance and promotes tumor migration and invasion, and thus the recurrence of the tumor [50].

Autophagy found to be associated with the progression of glioma, especially in HGG type and LC3B significantly correlated with poorer prognosis [51]. Accumulating research suggest that autophagy-related genes can act as oncogenes and result in poor prognosis [18]. Several recent studies report that, autophagy induction has been associated with tumor cell survival and adaptation to nutrient stress in GBM as well as radio-resistance of glioma stem cells [18, 52, 53]. This may reflect autophagy-mediated tumor survival under hypoxia and therapy-induced stress, maintaining glioma stem-like cells and promoting recurrence [50]. However, LC3B overexpression could also indicate defective autophagic flux, as static detection cannot distinguish increased autophagosome formation from impaired degradation [54]. Hence, elevated LC3B might represent either enhanced pro-survival autophagy or blocked flux—both linked to poor prognosis but via different mechanisms.

With multivariate regression analysis, the current study found that PD-L1 is an independent factor in relation to OS. High PD-L1 was considered as risk factor for mortality which agrees with previous studies [4, 35]; However, this contrasted with others [23, 37, 38, 55]. The conflict in results can be explained by the involvement of all grades of glioma in the study [23].

Also multivariate analysis revealed that adjuvant chemotherapy was a predictor for good prognosis. In line with these findings a previous study stated that prognosis of adult patients with GBM remains poor; however, adjuvant treatments improve progression-free and overall survival [56].

Recent findings from experiments with murine melanoma cells and human ovarian cancer cells indicated that cells with high levels of the PD-L1 receptor expression are more sensitive to autophagy inhibitors, as compared to cells that weakly express PD-L1 [57].

Our study is thought to be the first one that established a correlation between PD-L1 and LC3B expression in GBM. Also, no previous study studied these two molecules parallelly in the same time in GBM. We found significant correlation between PD-L1 and LC3B expression in GBM. These biological molecules have been associated with unfavorable prognostic value. The synergistic effect of both could strengthen their adverse prognostic role. While no previous study reported this specific relationship in GBM, other studies have shown links between autophagy and PD-L1 expression [58, 59] for example: autophagy was enhanced in PD-L1 positive gastric cancers [60] and mTORC1 can by direct intermediate between autophagy and PD-L1 pathway, it inhibits autophagy and it is also important signaling target for PD-L1 intracytoplasmic tail [61]. Also, PD-L1 expression in NSCLC is upregulated by oncogenic activation of the AKT-mTOR pathway and mTOR-mediated IFN-γ expression [62].

So PD-L1 overexpressing tumor cells may be more sensitive to autophagy inhibitors suggesting a potential therapeutic use. Further research, mechanistic and functional studies for understanding of these interactions will guide the potential use of autophagy inhibitors in combination with anti-PD-L1 therapy for enhancing antitumor response.

The main limitations of the current study are the retrospective study design and being conducted in single center together with the modest sample size, may limit the generalizability of the findings. Also molecular markers known to influence glioblastoma prognosis—such as IDH mutation status, MGMT promoter methylation, and ATRX expression—were not assessed, which restricts comprehensive molecular stratification. The evaluation of PD-L1 expression was based on single-biopsy immunohistochemistry, which may not fully capture the well-documented spatial and temporal heterogeneity of PD-L1 expression within glioblastoma. Future multi-institutional studies incorporating molecular profiling and multi-regional sampling are warranted to validate and expand these findings.

## Conclusion

In summary, the robust expression of PD-L1 in glioblastoma is associated with poor prognosis. Additionally, high expression of LC3B in GBM suggests increased autophagy activity. High PD-L1 and LC3B expression were associated with worse outcomes suggesting potential prognostic value and raise the hypothesis that concurrent targeting of autophagy and immune checkpoint pathways could provide therapeutic benefit in glioblastoma, warranting further preclinical and clinical investigation. However, further large-scale studies are needed to validate the potential value of PD-L1 and LC3B as prognostic markers and treatment targets for GBM patients.

## Data Availability

All data generated or analyzed during this study are included in this published article.
